# ERα-36 regulates progesterone receptor activity in breast cancer

**DOI:** 10.1186/s13058-020-01278-7

**Published:** 2020-05-19

**Authors:** Henri-Philippe Konan, Loay Kassem, Soleilmane Omarjee, Ausra Surmieliova-Garnès, Julien Jacquemetton, Elodie Cascales, Amélie Rezza, Olivier Trédan, Isabelle Treilleux, Coralie Poulard, Muriel Le Romancer

**Affiliations:** 1 Université de Lyon, F-69000 Lyon, France; 2 Inserm U1052, Centre de Recherche en Cancérologie de Lyon, Centre Léon Bérard, Bâtiment D, 28 rue Laennec, 69373 Lyon Cedex 08, F-69000 Lyon, France; 3 CNRS UMR5286, Centre de Recherche en Cancérologie de Lyon, F-69000 Lyon, France; 4 Clinical Oncology Department, Faculty of Medicine, Cairo University, Cairo, Egypt; 5 Cancer Research UK, Cambridge Institute, University of Cambridge, Cambridge, CB2 0RE UK; 6 genOway, 69007 Lyon, France; 7 Medical Oncology Department, Centre Léon Bérard, F-69000 Lyon, France; 8 Pathology Department, Centre Léon Bérard, F-69000 Lyon, France

**Keywords:** Breast cancer, ERα-36, Progesterone receptor, Transcription, Biomarker

## Abstract

**Background:**

Alterations in estrogen and progesterone signaling, via their respective receptors, estrogen receptor alpha (ERα) and progesterone receptor (PR), respectively, are largely involved in the development of breast cancer (BC). The recent identification of ERα-36, a splice variant of ERα, has uncovered a new facet of this pathology. Although ERα-36 expression is associated with poor prognosis, metastasis development, and resistance to treatment, its predictive value has so far not been associated with a BC subtype and its mechanisms of action remain understudied.

**Methods:**

To study ERα-36 expression in BC specimens, we performed immunochemical experiments. Next, the role of ERα-36 in progesterone signaling was investigated by generating KO clones using the CRISPR/CAS9 technology. PR signaling was also assessed by proximity ligation assay, Western blotting, RT-QPCR, and ChIP experiments. Finally, proliferation assays were performed with the IncuCyte technology and migration experiments using scratch assays.

**Results:**

Here, we demonstrate that ERα-36 expression at the plasma membrane is correlated with a reduced disease-free survival in a cohort of 160 BC patients, particularly in PR-positive tumors, suggesting a crosstalk between ERα-36 and PR. Indeed, we show that ERα-36 interacts constitutively with PR in the nucleus of tumor cells. Moreover, it regulates PR expression and phosphorylation on key residues, impacting the biological effects of progesterone.

**Conclusions:**

ERα-36 is thus a regulator of PR signaling, interfering with its transcriptional activity and progesterone-induced anti-proliferative effects as well as migratory capacity. Hence, ERα-36 may constitute a new prognostic marker as well as a potential target in PR-positive BC.

## Background

Breast cancer (BC) is the most common cancer among women worldwide. More than 75% of breast tumors express the estrogen receptor α (ERα) in the nucleus and predominantly belong to the luminal subtype. ERα plays a major role in BC tumorigenesis as it regulates cell cycle, cell survival, and angiogenesis [1]. Interfering with the ERα pathway using anti-estrogens (either selective estrogen receptor modulators, such as tamoxifen, or selective estrogen downregulators, such as fulvestrant) or through estrogen deprivation (e.g., aromatase inhibitors) increases the survival of ERα-positive BC patients. Despite the high level of sensitivity of luminal tumors to endocrine therapy, treatment efficacy is limited by intrinsic and acquired resistance [2, 3]. Indeed, 30–50% of patients relapse in the adjuvant setting and eventually die following the development of metastases [2, 4]. More recently, ERα-36, a splice variant of ERα, was identified as a novel actor of breast tumorigenesis. ERα-36 is encoded by the *ESR1* locus, transcribed from a promoter located in the first intron, resulting in a shortened receptor. ERα-36 retains the DNA-binding domain, but lacks both transactivation domains, AF-1 and AF-2. Furthermore, the last 138 amino acids are replaced by a unique 27 amino acid sequence at the C-terminal domain [5]. Compared to ERα, ERα-36 displays distinct expression patterns. Indeed, while ERα is mainly expressed in the nucleus of ERα-positive tumors, ERα-36 is mainly expressed at the level of the plasma membrane of breast tumor cells [6], co-localized with caveolin, a typical cell surface protein [7, 8]. ERα-36 was shown to activate ERK1/2 through the protein kinase C delta signaling pathway, leading to an increase in the expression of cyclin D1/CDK4, which increases cell cycle progression [9]. In addition, binding of ERα-36 to ERK prevents its dephosphorylation by MKP3 and enhances a paxillin/cyclin D1 pathway [10]. Moreover, ERα-36 signaling contributes to the potential invasion and metastatic spread of cancer cells by upregulating aldehyde dehydrogenase 1A1 [11]. Surprisingly, unlike ERα, ERα-36 is activated by the estrogen antagonist tamoxifen and fulvestrant, both compounds routinely used in ERα-positive BC treatment [8]. Accumulating experimental and clinical evidence supports that BC may arise from mammary stem/progenitor cells which possess self-renewal abilities. Recently, it was reported that ERα-36-mediated estrogen signaling plays an important role in the maintenance of ERα-positive and ERα-negative breast cancer stem/progenitor cells [12]. Moreover, overexpression of ERα-36 in normal mammary epithelial cells causes loss of adhesion, enhanced migration, and resistance to apoptosis [13].

ERα-36 is also a marker of poor prognosis in BC, and its expression is associated with resistance to tamoxifen treatment, probably due to its high expression in stem cells, known to possess intrinsic resistance to treatment [11, 14].

The aim of this study was to investigate whether the prognostic value of ERα-36 was associated with a particular subtype of BC. We unveiled a correlation between ERα-36 expression and poorer PR-positive patient survival, suggesting a functional relationship between ERα-36 and PR signaling. We clearly showed that ERα-36 modulates PR expression and activity, regulating cell proliferation, thus confirming its importance in BC.

## Methods

### Cell culture

T47D were cultured in RPMI-1640 medium, supplemented with 10% fetal bovine serum (FBS), 2% penicillin-streptomycin (Life Technologies), and insulin (10 μg/ml). Cos7 cells were maintained in DMEM, supplemented with 10% FBS and 2% penicillin-streptomycin (Life Technologies). All cell lines were grown in a humidified atmosphere with 5% CO_2_ at 37 °C, authenticated by Eurofins and tested for *Mycoplasma* infection (Lonza, Rockland, ME, USA).

Prior to experiments, when it was indicated, cells were grown in phenol red-free medium supplemented with 10% charcoal-stripped serum (Biowest). Cells were then treated with 10 nM of R5020 (Perkin Elmer) or E2 (Sigma) for the indicated times.

### Generation of CRISPR ERα-36 KO cell lines

#### Electroporation of T47D cells

Cells were grown at subconfluence and electroporated with CRISPR reagents after cell dissociation using the Neon electroporator Invitrogen 1750 V-20 ms-1pulse. Electroporated cells were cultured as single cells to obtain pure clonal populations.

#### Strategy

Guide RNAs were designed using an in-house genOway’s tool, and those with the highest score were selected.

Targeted sequences were as follows: #1: 5′ TTAATAAGTACACACCGCAG AGG 3′; #2: 5′ CTGTGAGGCCTTATGACCAG AGG 3′.

These guide RNAs were designed to induce the deletion of an ERα-36-specific sequence by cutting into intron 8 and downstream of exon 9 (intron 31 and exon 32 with genOway’s numbering). ERα-36 isoform-specific knock-out clones were amplified, and isolated DNA was characterized by PCR amplification, as the 393-bp deleted sequence includes the ERα-36-specific exon 9 splice acceptor site, coding sequence, and STOP codon.

#### Antibodies

**Table:** Information of primary antibodies

Antibodies	Supplier	Origin	Dilution for WB	Dilution for PLA	Dilution for IHC
ERα-36	Homemade	Rabbit	1/1000	1/100	1/50
PR (AB8)	Thermo Scientific	Mouse		1/500	
PR (H190)	Santa Cruz Biotechnology	Rabbit	1/2000		
p-PRS294	Cell Signaling Technology	Rabbit	1/1000	1/500	
p-PR S345	Cell Signaling Technology	Rabbit	1/1000	1/500	
Tubulin	Sigma	Mouse	1/10000		
V5-tag (D3H8Q)	Cell Signaling Technology	Rabbit	1/1000		
Flag M2	Sigma	Mouse	1/1000		
p44/42 MAPK (Erk1/2)	Cell Signaling Technology	Rabbit	1/1000		
Phospho p44/42 MAPK (Erk1/2)	Cell Signaling Technology	Rabbit	1/1000		

#### Luciferase reporter assay

HeLa cells (7.5 × 10^4^) were plated in 24-well plates 24 h prior to transfection. The transfected DNA included 100 ng of reporter plasmid and 25 ng of pRL-TK Renilla luciferase vector (Promega) used as an internal control, together with various amounts of expression vectors, as indicated. Total transfected DNA was kept constant by adding empty pSG5-Flag vectors. The cells were induced with 10 nM R5020 24 h following transfection, then harvested after an additional 24 h and assayed for luciferase activity following the manufacturer’s instructions. Luciferase activities were normalized against the activity of the internal control Renilla luciferase.

#### Immunofluorescence

T47D cells (2 × 10^5^) were grown on coverslips in 12-well plates. After treatment, cells were fixed in methanol for 2 min and washed twice in PBS. Non-specific binding was blocked using a 1% gelatin solution for 30 min at room temperature. Cells were incubated with PR antibody for 1 h at 37 °C, subsequently with the secondary antibodies Alexa Fluor 488 anti-mouse (Jackson ImmunoResearch, Cambridge, UK) (1:2000e) and Alexa Fluor 568 anti-rabbit (Invitrogen, Carlsbad, USA) (1:1000e) in Dako diluent for 1 h. Finally, coverslips were mounted on glass slides in mounting solution (Dako, Carpinteria, CA, USA). The fluorescent slides were viewed under the Nikon NIE microscope.

#### Immunoprecipitation and Western blot analysis

Cells were lyzed using RIPA buffer (50 mM Tris HCl, pH 8, 150 mM NaCl, 1 mM EDTA, 1% NP-40, and 0.25% deoxycholate) supplemented with protease inhibitor tablets (Roche Molecular Biochemicals) and phosphatase inhibitors (1 mM NaF, 1 mM Na_3_VO_4_, and 1 mM β-glycerophosphate). Protein extracts were incubated with primary antibodies overnight at 4 °C on a shaker. Protein G-Agarose beads were added, and the mixture was incubated for 2 h at 4 °C. The immunoprecipitated proteins were separated by sodium dodecyl sulfate-polyacrylamide gel electrophoresis (SDS-PAGE) and analyzed by Western blot, then visualized by electrochemiluminescence (ECL, Roche Molecular Biochemicals).

#### Chromatin immunoprecipitation

ChIP experiments were performed according to the manufacturer’s protocol (SingleChIP enzymatic chromatin IP Kit - Cell signaling) with antibodies against PR, ERα, and IgG. Results are expressed relative to the signal obtained with chromatin input. Primer sequences are indicated in the Additional file 1.

#### RNA extraction and real-time RT-qPCR analysis

Total RNA (1 μg) was extracted and purified using TRI Reagent (Sigma-Aldrich, USA), prior to being reverse-transcribed using 100 ng of random primers following the Superscript II (Thermo Fisher, USA) protocol. Real-time PCR was performed with SYBR Green qPCR master mix (BioRad) in a Step One plus real-time PCR detection system (Applied Biosystems). All amplifications were performed in triplicate. Mean values of triplicate measurements were calculated according to the −ΔΔCt quantification method and were normalized against the expression of 28S ribosomal mRNA as reference. Data were presented as mean ± SEM. Sequences of the oligonucleotides used are listed in Additional file 2.

#### Proximity ligation assay, image acquisition, and analysis

This technology exposes protein/protein interactions in situ [15]. Briefly, cells were seeded and fixed with cold methanol. After saturation, the different couples of primary antibodies were incubated for 1 h at 37 °C. The proximity ligation assay (PLA) probes consisting of secondary antibodies conjugated with complementary oligonucleotides were incubated for 1 h at 37 °C. The amplification step followed the ligation of nucleotides for 100 min at 37 °C. Samples were subsequently analyzed under fluorescence microscopy.

The hybridized fluorescent slides were viewed under a Nikon Eclipse Ni microscope. Images were acquired under identical conditions at × 60 magnification. Image acquisition was performed by imaging DAPI staining at a fixed Z Position while a Z stack of ± 5 μm at 1 μm intervals was carried out. The final image was stacked to a single level before further quantification. On each sample, at least one hundred cells were counted. Analysis and quantification of these samples were performed using the ImageJ software (free access). PLA dots were quantified on 8-bit images using the “Analyse Particles” command, while cells were counted using the cell counter plugin.

IHC images were also acquired using a Nikon Eclipse Ni microscope at × 40 magnification, and PLA dots were quantified as described above.

#### Glutathione transferase pull-down assay

ERα-expressing plasmids were transcribed and translated in vitro using T7-coupled reticulocyte lysate in the presence of [^35^S] methionine. Glutathione transferase (GST) fusion proteins were incubated with labeled proteins in 200 μl of binding buffer (Tris 20 mM pH 7.4, NaCl 0.1 M, EDTA 1 mM, glycerol 10%, Igepal 0.25% with 1 mM DTT and 1% milk) for 2 h at room temperature. After washing, bound proteins were separated by SDS-PAGE and visualized by autoradiography.

#### Proliferation studies

4 × 10^3^ cells seeded onto a 96-well plate were plated 5 h before incubation with the different hormones (E2, R5020, or ethanol). Images were acquired using an IncuCyte ZOOM over 7 days, and cell proliferation was measured as the percentage of cell density observed over this period. Results are represented as graphs indicating the rate of proliferation over time, extrapolated from at least three independent experiments, each performed in triplicate.

#### Wound healing assay

Cells were plated in duplicate in 6-well plates and grown to confluence. Wounds were then performed with a p200 pipette tip. After washes to remove cellular debris, three images of each well were taken. The width of the wound was measured at 3 places and recorded as *t* = 0. The cells were then allowed to migrate back into the wounded area. After 16 h, the width of the open area was measured. Cell migration was expressed as the percentage of the gap (*t* = 16) relative to the primary width of the open area (*t* = 0). Images were acquired on a phase contrast microscope (Zeiss, Axiovert 25). All experiments were performed in triplicate.

#### Patient population

We screened 200 consecutive female patients with operable breast cancers who had undergone radical surgery and received adjuvant/neoadjuvant therapy in the Centre Léon Bérard between January 1999 and December 2001. Paraffin blocks of tumor tissue were available for 182 patients. Among these, we failed to assess ERα-36 in 22 tumor specimens as a result of insufficient tumor or tissue loss during TMA preparation. Therefore, a total of 160 specimens were analyzed in this study.

Patients underwent radical surgery (either modified radical mastectomy (MRM) or breast-conserving surgery (BCS) with axillary lymph node (LN) staging). ERα-66 and PR were detected by immunohistochemistry, and tumors were considered positive if they display nuclear staining in 10% or more of the tumor cells. HER2 expression was determined using immunohistochemistry, and tumors were considered positive if they reached 3+ staining by immunohistochemistry or 2+ staining with HER2 amplification detected by FISH.

The data exported from patient files for analysis included age, histological subtype, maximum tumor size, number of LNs involved, SBR grade, ER, PR, HER2 status, date of diagnosis, date of relapse, and date of death or last clinical visit. Tumor samples and clinical data were obtained with the approval of the Institutional Review Board. This study is reported according to the REMARK criteria [16].

#### Immunohistochemical analysis

The breast tumor samples were inserted as triplicates using a 600-μm needle into 4 tissue microarray (TMA) blocks. The blocks containing invasive carcinoma were sectioned at a thickness of 4 μm. After deparaffinization and rehydration, endogenous peroxidases were blocked by incubating the slides in 5% hydrogen peroxide in sterile water. For heat-induced antigen retrieval, tissue sections were boiled in 10 mM citrate buffer pH 6.0 (Dako, Trappes, France) using a water bath at 98 °C for 50 min.

The slides were then incubated at room temperature for 1 h with the antibodies against ERα-36 (rabbit polyclonal antibody). These antibodies were diluted using an antibody diluent solution (Chemmate, Dako, Trappes, France) at 1/50. After rinsing in PBS, the slides were incubated with a biotinylated secondary antibody bound to a streptavidin peroxidase conjugate (LSAB+ Kit, Dako, Trappes, France). Bound antibodies were detected by adding the substrate 3,3-diamino-benzidine. Sections were counterstained with hematoxylin.

Blinded to the clinical data, biomarker expression was evaluated by 2 observers who assessed both the percentage and the intensity of the membranous staining for ERα-36 in the infiltrative carcinomatous cells only (faint cytoplasmic staining which was found in almost all malignant cells was not considered).

For scoring purposes, the highest intensity of staining in malignant cells was divided into 3 levels (0, no staining; 1, weak staining; 2, moderate to strong staining), and the percentage of stained cells was also classified into 3 levels (0, no stained cells; 1, staining in less than half of the malignant cells; 2, staining in half or more of the malignant cells). Then, both intensity and percentage scores were added to obtain a single score (from 0 to 4) in a manner similar to the Allred score for ER and PR staining [17]. For the purpose of correlation and survival analyses, tumors were considered to have a low expression for ERα-36 if they scored between 0 and 2 and were considered to have high expression above 2.

### Statistical analysis

The correlation between ERα-36 expression and clinico-pathologic characteristics was determined using Pearson’s chi-square test (or Fisher’s exact test). Distant metastasis-free survival (DMFS) was defined as the time from the date of histological diagnosis of breast cancer to the date of distant metastasis or death. Disease-free survival (DFS) was defined as the time from the date of histological diagnosis of breast cancer to the date of any cancer recurrence (local, distant, or contralateral) or death. Overall survival (OS) was defined as the time from the date of histological diagnosis of breast cancer to the date of death. The database was locked at 12 years of follow-up, and patients who were event-free at the last follow-up visit (or at 12 years) were censored.

Survival curves, median DMFS, DFS, and OS (if reached) in addition to 8-year DMFS, DFS, and OS (with 95%CIs) were derived from Kaplan-Meier estimates, and the curves were compared using log-rank test. Hazard ratios and 95%CIs were calculated using Cox regression model. Cox multivariate analysis was performed to determine whether a factor is an independent predictor of DMFS, DFS, or OS after adjusting for other significant factors at the univariate level. All statistical tests were two-sided, and the *p* value was considered statistically significant if inferior to 5%. Statistical analyses were performed using SPSS 20.0 statistics package.

## Results

### Clinico-pathological characteristics

We evaluated ERα-36 expression in a cohort of patients displaying invasive breast cancer using our polyclonal antibody specifically recognizing the ERα-36 isoform, which has already been validated for IHC experiments [10]. Table 1 summarizes the clinico-pathological characteristics of the patient cohort tested. For the 160 assessable patients, the median follow-up interval was 10 years (ranging from 0.2 to 12 years). Median age at diagnosis was 56.9 years (ranging from 30.4 to 87.4 years). Regarding tumor stage, 57.5% of patients displayed tumors exceeding 20 mm, and 52.5% had axillary lymph node (LN) metastases. 16.3% of the patients had SBR grade I tumors, 44.4% had grade II tumors, and 39.4% grade III tumors. Adjuvant chemotherapy was administered to 63.1% of patients, while 83.1% received adjuvant hormonal therapy.

**Table 1:** Clinico-pathological characteristics, treatment received, and ERα-36 expression in the patient cohort tested (160 patients)

Characteristic		Number	Percent
Age group (years)	< 50	51	31.9
	> 50	109	68.1
Menopausal status	Pre	57	35.6
	Post	103	74.4
Tumor size (cm)	< 2	68	42.5
	> 2	92	57.5
Axillary LN metastasis	No	76	47.5
	Yes	84	52.5
SBR grade	I	26	16.3
	II	71	44.4
	III	63	39.4
ERα-66 status	Negative	14	8.8
	Positive	145	90.6
	Missing	1	
PR status	Negative	40	25.3
	Positive	118	74.7
	Missing	2	
HER2 status	Negative	129	84.9
	Overexpressed	23	15.1
	Missing	8	
Breast cancer subtype	Luminal	142	91.6
	Basal	10	6.5
	HER2 driven	3	1.9
	Missing	5	
Adjuvant Hormonal treatment	No	27	16.9
	Yes	133	83.1
Adjuvant (or neoadj) chemotherapy	No	59	36.9
	Yes	101	63.1
ERα-36	Low	95	59.4
	High	65	40.6

### Pattern of ERα-36 expression

**Fig. 1:**
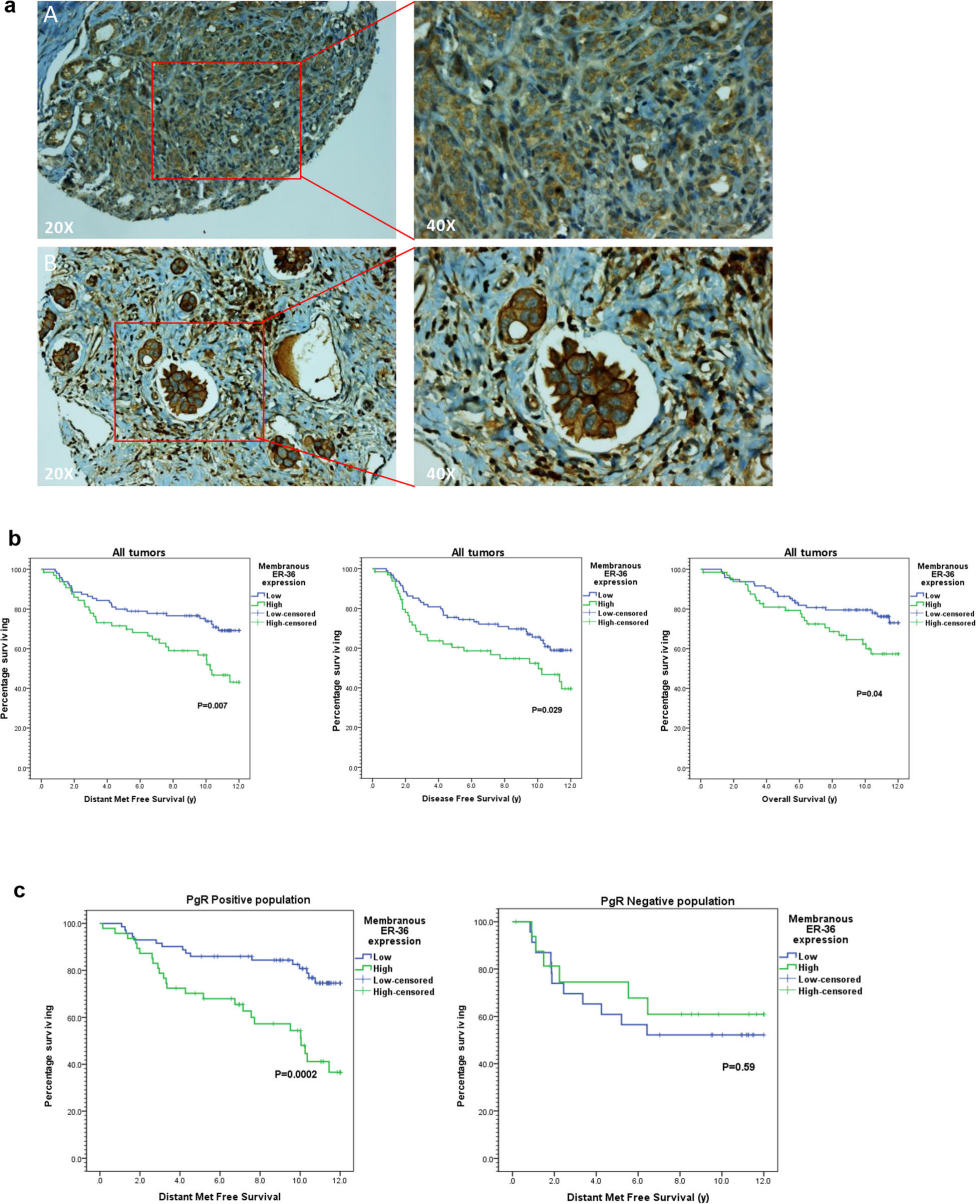
Expression of ERα-36 in breast tumors. **a** ERα-36 expression was analyzed by immunohistochemistry (IHC) on formalin-fixed human tumors. Representative images of different IHC staining profiles are shown (A: low expression; B: high expression). **b** Kaplan-Meier estimates of disease-metastases free survival (DMFS) (left), disease-free survival (DFS) (middle), and overall survival (OS) (right) in patients with low (blue) versus high (green) membranous ERα-36 expression. **c** Kaplan-Meier estimates of DMFS in patients with low (blue) versus high (green) ERα-36 expression in 2 groups of patients according to PR expression

Regarding the immunohistochemical (IHC) analysis of ERα-36, most of the tumors displayed a faint diffuse cytoplasmic ERα-36 expression (Fig. 1a), which for the purpose of statistical analysis was discarded. However, only 65 tumors (40%) had a high membrane expression, while 95 (60%) had a low or were devoid of membrane expression (Fig. 1a). The correlation between ERα-36 expression and different clinico-pathological parameters was then statistically investigated (Table 2). No significant association was observed between high ERα-36 and age, menopausal status, tumor size, ERα-66 status, PR status, and axillary lymph node metastasis, except for high SBR grade (grade III) (*p* = 0.04).

**Table 2:** Correlation between ERα-36 expression and clinico-pathological features

Variable		ERα-36 low, no. (%)	ERα-36 high, no. (%)	*p* value
Age (years)	Mean (+ SD)	56.6 (+ 12.3)	57.9 (+ 12.9)	0,43^†^
Age groups	< 50 years	32 (33.7%)	19 (29.2%)	0.5
	> 50 years	63 (66.3%)	46 (70.8%)	
Side	Right	42 (44.2%)	30 (46.2%)	0.8
	Left	53 (55.8%)	35 (53.8%)	
T. size	< 2 cm	41 (43.2%)	27 (41.5%)	0.83
	> 2 cm	54 (56.8%)	38 (58.5%)	
LN met	Negative	45 (47.4%)	31 (47.4%)	0.9
	Positive	50 (52.6%)	34 (52.3%)	
SBR grade	Gr 1	13 (13.7%)	13 (20%)	**0.039**^††^
	Gr 2	50 (52.6%)	21 (32.3%)	
	Gr 3	32 (33.7%)	31 (47.7%)	
ERα status	Negative	9 (9.6%)	5 (7.7%)	0.7^††^
	Positive	85 (90.4%)	60 (92.3%)	
PR status	Negative	23 (24.5%)	17 (26.6%)	0.7
	Positive	71 (75.5%)	47 (73.4%)	
ERα/PR detailed	ERα+/PR+	71 (75.5%)	47 (73.4%)	0.78^††^
	ERα+/PR−	14 (14.9%)	12 (18.8%)	
	ERα−/PR+	0 (0.0%)	0 (0.0%)	
	ERα−/PR−	9 (9.6%)	5 (7.8%)	
Her 2 status	Negative	78 (85.7%)	51 (83.6%)	0.7
	Over-expressed	13 (14.3%)	10 (16.4%)	

*Tam* tamoxifen, *AI* aromatase inhibitor, *Anthra* anthracycline

*Correlations tested using Pearson’s chi-square test (2 sided) unless otherwise specified

^†^Difference between means using the Student *t* test

^††^Fisher’s exact test

### High ERα-36 expression predicts poorer outcome in BC

At 8 years, rates of distant metastasis-free survival (DMFS), disease-free survival (DFS), and overall survival (OS) were all poorer in patients with high compared to low ERα-36 expression, with 59.0% versus 76.6% (DMFS: HR = 2.02, 95%CI 1.2–3.4, *p* = 0.007), 54.7% versus 70.9% (DFS: HR = 1.69, 95%CI 1.1–2.7, *p* = 0.029), and 68.6% versus 79.6% (OS: HR = 1.82, 95%CI 1.02–3.2, *p* = 0.040), respectively (Fig. 1b). In the multivariate analysis, when adjusted to tumor size, LN metastasis, and SBR grade (other significant prognostic factors in the univariate model), high ERα-36 expression was still an independent predictor of poorer DMFS (HR = 1.93, 95%CI 1.1–3.3, *p* = 0.016) with a tendency towards poorer OS (HR = 1.65, 95%CI 0.9–3.0, *p* = 0.09). In addition to ERα-36, large tumor size (HR = 1.84, 95%CI 1.04–3.28, *p* = 0.04) and high SBR grade (HR = 2.04, 95%CI 1.2–3.5, *p* = 0.008) were also an independent predictor of poor DMFS in the same multivariate model.

Interestingly, the impact of ERα-36 expression on distant metastasis-free relapse was limited to PR-positive patients (Fig. 1c). Indeed, in the PR-positive patient cohort, the 8-year DMFS rate was 57.2% in patients with high ERα-36 expression compared to 84.7% in patients with low ERα-36 expression (*p* < 0.001). On the contrary, in the PR-negative cohort, the difference in this rate was non-significant with values of 60.7% and 52.2% in high versus low ERα-36 expression (*p* = 0.59), respectively.

Altogether, these results show that ERα-36 expression at the plasma membrane is a poor prognostic marker impacting survival of PR-positive BC patients.

### ERα-36 is a new partner of PR

**Fig. 2:**
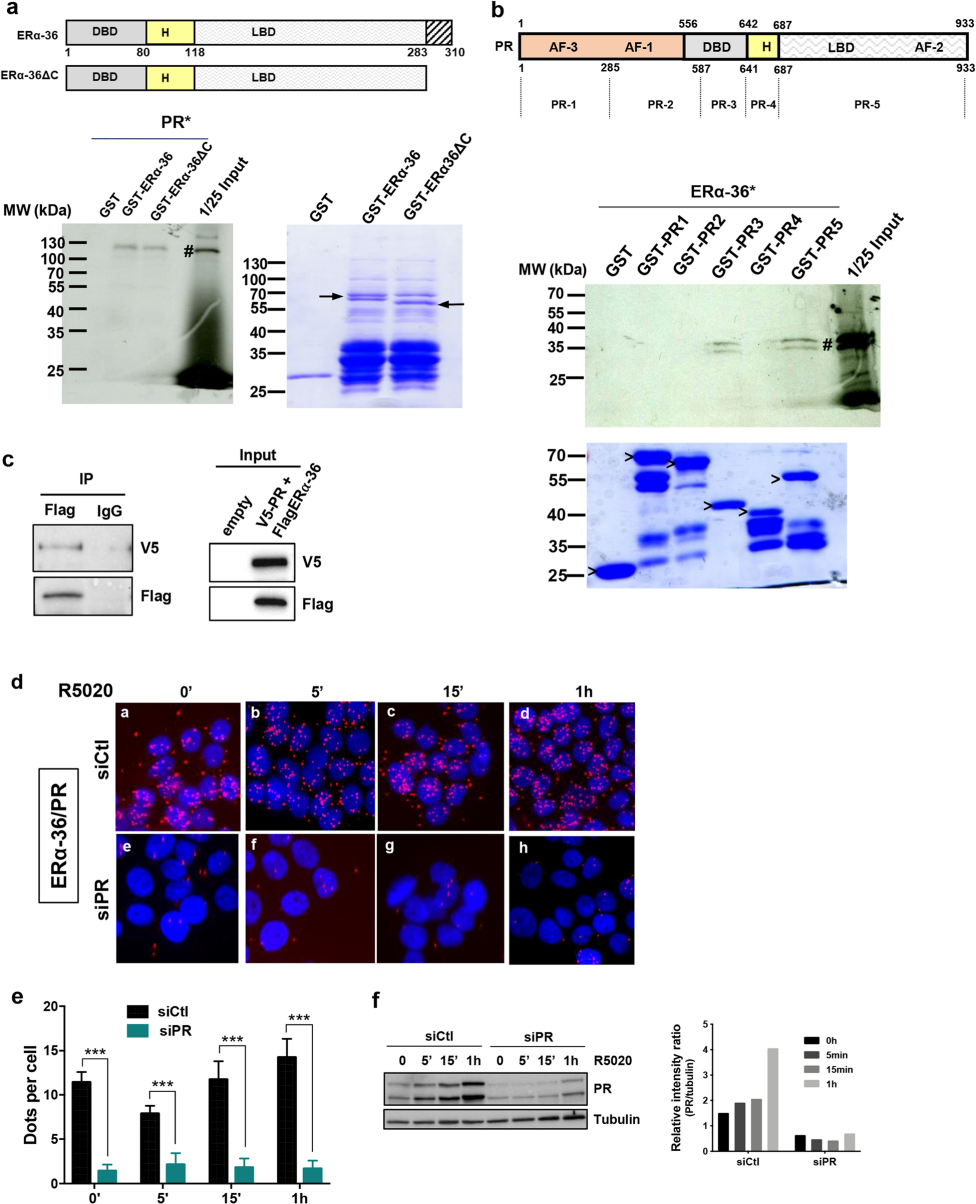
ERα-36 interacts with PR. **a** A radioactive GST pull-down assay was performed by incubating in vitro ^35^S-labeled PR (PR #) with GST, GST-ERα-36, and GST-ERα-36ΔC. The corresponding Coomassie-stained gel is shown below. Arrows indicate the full-length fusion proteins. **b** PR was divided into 5 fragments (PR1 to PR5). Radioactive ERα-36 (ERα-36 #) was incubated with the different domains of PR fused to GST, and the bound proteins were visualized by autoradiography. The corresponding Coomassie-stained gel is shown below. “>” indicates the full-length fusion proteins. **c** pSG5Flag-ERα-36 and pCDNA3V5-PR were overexpressed in Cos7 cells. Cell lysates were immunoprecipitated with the anti-Flag antibody, and the presence of ERα-36 and PR was visualized by Western blot using the anti-Flag and anti-V5 antibodies, respectively. The lower panel shows the expression of the different proteins in the input. **d** Proximity ligation assay (PLA) was used to detect the cellular co-localization of endogenous ERα-36 and PR in T47D, grown on coverslips in 12-well plates. Cells were transfected with control siRNA (siCtl) or with siRNA against PR (siPR) and treated for the indicated times with 10 nM of R5020. PLA for ERα-36/PR interaction was performed with anti-PR- and anti-ERα-36-specific antibodies. The nuclei were counterstained with DAPI (blue) (Obj, × 60). The detected interactions are represented by red dots. **e** Quantification of the number of signals per cell was performed using computer-assisted analysis, as reported in the “Methods” section. The mean ± SD of one experiment representative of three experiments is shown. **f** The efficacy of PR siRNA treatment analyzed by Western blot analysis is shown in the left-hand panel and quantified in the right-hand panel where the PR expression relative to tubulin was quantified using ChemiDoc MP (Biorad)

Based on the previous results, we hypothesized that ERα-36 could be a bad prognostic marker in PR-positive BC because it interferes with progesterone signaling. To evaluate whether these proteins interacted directly, we initially conducted a GST pull-down experiment. We found that radioactive PR specifically interacts with the full length ERα-36 and its truncated form ERα-36ΔC (truncated C-terminal part), but not with the GST (Fig. 2a). By separating the PR protein into 5 fragments (PR1 to PR5), we further identified PR3 and PR5 fragments, as the sites of ERα-36/PR interaction (Fig. 2b). We also validated the interaction by co-immunoprecipitation after transfection of both proteins into Cos7 cells (Fig. 2c). In addition, we investigated this interaction and its localization in T47D cells that express both proteins, using proximity ligation assay (PLA) and specific antibodies. The images obtained revealed red dots in the nucleus of T47D cells, illustrating the interaction of ERα-36 with PR independently of progesterone treatment (Fig. 2d–f). The downregulation of PR silencing its expression was performed to validate the specificity of the ERα-36/PR interaction.

In conclusion, this nuclear interaction suggests that ERα-36 could regulate PR transcriptional activity.

### ERα-36 regulates PR expression

**Fig. 3:**
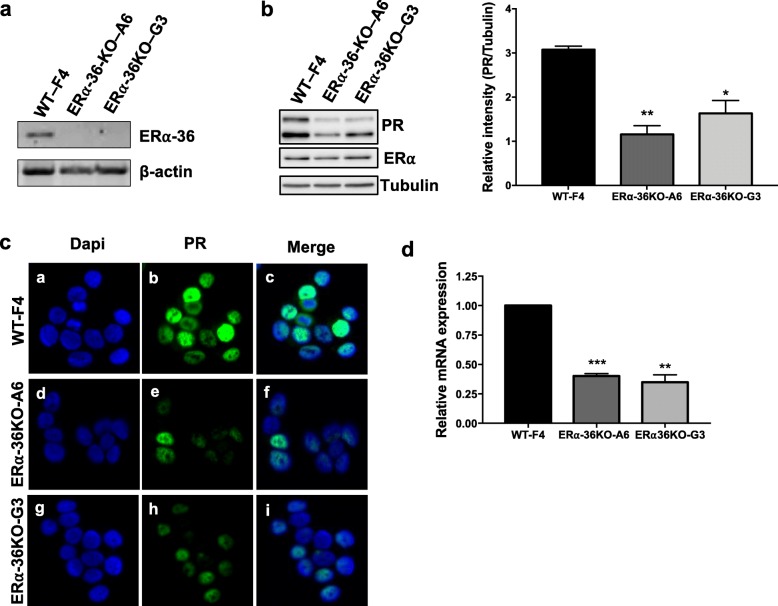
ERα-36 regulates PR expression. **a** ERα-36 and actin mRNA expression were analyzed by RT-PCR in T47D clones: F4 (WT) and A6 (KO-ER36) and G3 (KO-ERα-36). **b** PR, ERα, and tubulin expression were assessed by Western blot in the three clones. Western blot quantification was determined comparing PR to tubulin using ChemiDoc MP (Biorad) to measure the chemiluminescence from the immunoblots. The values represent the mean ± SEM of three independent experiments. The *p* value was determined comparing each ERα-36 KO clones to the WT using Student’s *t* test. *p < 0.05, **p < 0.01. **c** PR expression was studied by immunofluorescence in the 3 clones. The nuclei were counterstained with DAPI (blue) (Obj, × 40). **d** Total RNA was prepared and cDNAs analyzed by RT-qPCR with specific primers for PR. The values were normalized against 28S mRNA and represent the mean ± SEM of three experiments. The *p* value was determined comparing each ERα-36 KO clone to WT cells using Student’s *t* test. **p < 0.01, **p < 0.001

To further investigate the role of ERα-36 in progesterone signaling, we used the CRISPR/CAS9 technology to specifically knock out the exon coding the isoform. Genomic DNA sequencing of ERα-36 KO clones of T47D cells revealed deletion at the targeted site. By RT-PCR, we evaluated the mRNA expression of the different clones and chose F4 for wild type (WT) and A6 and G3 for KO clones (Fig. 3a). Next, we assessed the PR expression by Western blot and observed a significant decrease in KO cells compared to WT cells (Fig. 3b). This effect was not due to a decrease in ERα expression, as evidenced by the Western blot (Fig. 3b). We also studied PR localization, which revealed that although the staining decreased in KO compared to WT clones, it remained within the nucleus (Fig. 3c). We then confirmed that this decrease in PR expression occurred in KO cells at the mRNA level by RT-qPCR (Fig. 3d). We hypothesized that this decrease could be due to a defect of ERα binding to the PR promoter, but the low level of ERα binding on the chromatin did not allow us to conclude (Additional file 3).

### ERα-36 regulates PR signaling

**Fig. 4:**
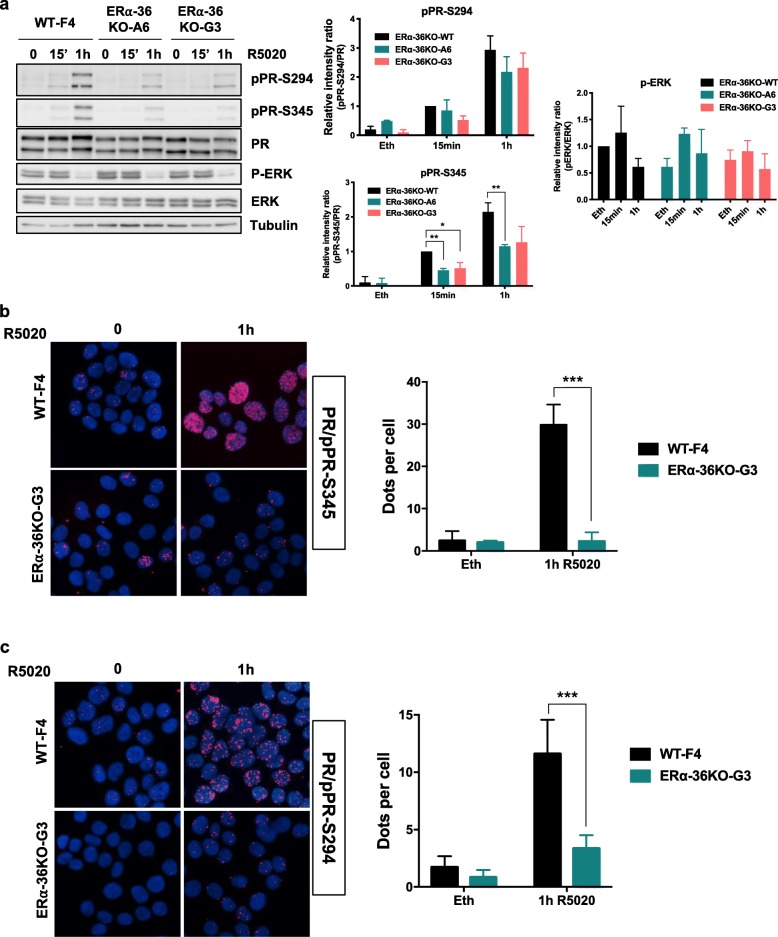
ERα-36 regulates PR signaling. **a** WT and KO ERα-36 cells were treated with R5020 for the indicated times; cell extracts were then loaded onto a gel and assessed by Western blot for PR, pPR-S294 and S345, P-ERK, ERK, and tubulin expression. Quantification was performed in the right-hand panels. **b** Phosphorylation of PR on S345 was also studied by PLA using a specific antibody and an antibody recognizing PR in the WT and the KO ERα-36 G3 clones. Quantification of the results is shown in the right-hand panel. **c** The same experiment was performed as in **b** to measure the PR phosphorylation on S294

Next, we investigated whether ERα-36 regulates progesterone signaling. First, we treated T47D cells with R5020, the progesterone analog for different periods of time and performed Western blot analyses to measure the ERK activation and PR phosphorylation on its main sites: S294 and S345. As shown in Fig. 4a, although pPR S294 and S345 decreased in KO ERα-36 clones compared to the WT, P-ERK remained activated. Quantification of the pPR/PR ratio showed that ERα-36 KO reduced the phosphorylation of PR. Treating T47D cells with an ERK inhibitor showed that ERK activity was partially implicated in the phosphorylation of PR (Additional file 4). The decrease in pPR was confirmed by PLA, showing that both pPR in the nucleus are lower in KO clones compared to the WT T47D cells (Fig. 4b, c).

**Fig. 5:**
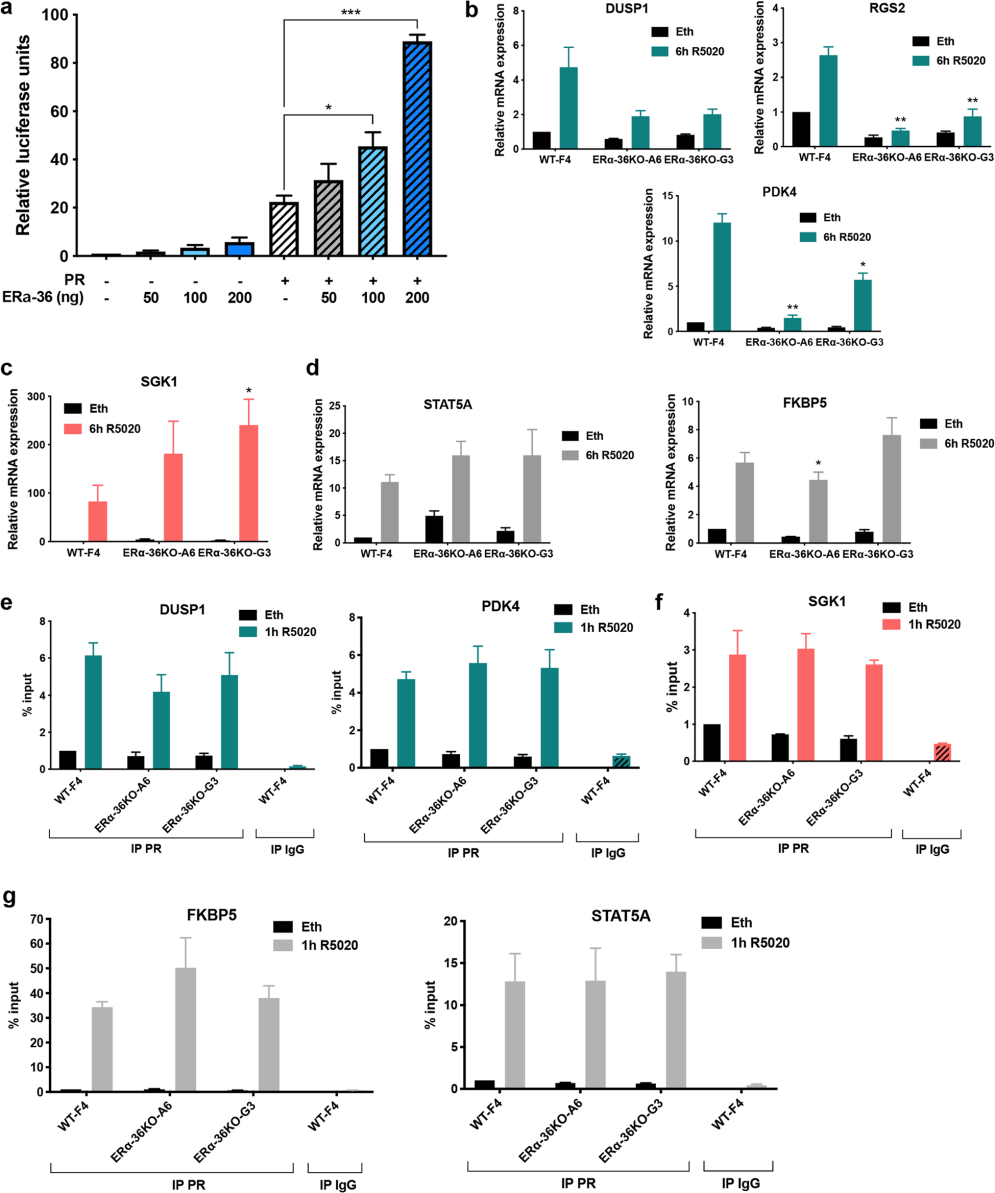
ERα-36 regulates PR transcriptional activity. **a** HeLa cells were transiently transfected with MMTV-LUC reporter plasmid and expression vectors encoding PR (10 ng) and ERα-36 (from 50 to 200 ng) using Lipofectamine 2000. Transfected cells were grown in a hormone-free medium for 48 h in the presence or absence of 10 nM R5020, and extracts of the harvested cells were tested for luciferase activity using the Promega luciferase assay kit. The results were normalized as indicated and presented as the mean ± SEM of at least three independent experiments. The *p* value was determined using Student’s *t* test. *p < 0.05, ***p < 0.001. **b**–**d** Clones of T47D were treated, or not (Eth), 6 h with 10 nM of R5020. Total RNA was prepared and cDNAs analyzed by RT-qPCR with specific primers for SGK1, STAT5A, FKBP5, PDK4, DUSP1, and RGS2. The values were normalized against 28S mRNA and represent the mean ± SEM of three experiments. The *p* value was determined comparing each ERα-36 KO clones to the corresponding condition in the WT using Student’s *t* test. **p < 0.01, ***p < 0.001. **e**–**g** T47D clones, grown in a charcoal-stripped serum for 48 h and then treated with 10 nM R5020 for 1 h, were subjected to ChIP assay using an anti-PR antibody. The precipitated DNA fragments were used for qPCR analysis using specific primers for the indicated promoters. The results are expressed relative to the signal obtained from input chromatin. The mean ± SEM of at least three experiments is shown. The *p* value determined by comparing each ERα-36 KO clone to the corresponding condition in WT cells using Student’s *t* test was not significant

We also assessed whether ERα-36 regulates PR transcriptional activity, by performing reporter luciferase assays. We found that ERα-36 stimulates PR activity in a dose-dependent manner (Fig. 5a). Next, we verified the expression of known PR target genes, which appeared to be downregulated (Fig. 5b and Additional file 5), upregulated (Fig. 5c), or unchanged (Fig. 5d) in ERα-36 KO clones compared to the WT clone. To evaluate whether these variations in gene expression were related to a change in PR binding, we performed ChIP experiments with PR antibody and found that PR binding to chromatin was not significantly modified in KO ERα-36 versus WT clones for all genes targeted irrespective of their level of expression (Fig. 5e–g).

In conclusion, ERα-36 regulates the PR transcriptional activity independently of PR binding to chromatin.

### ERα-36 regulates progesterone-mediated cell proliferation and migration

**Fig. 6:**
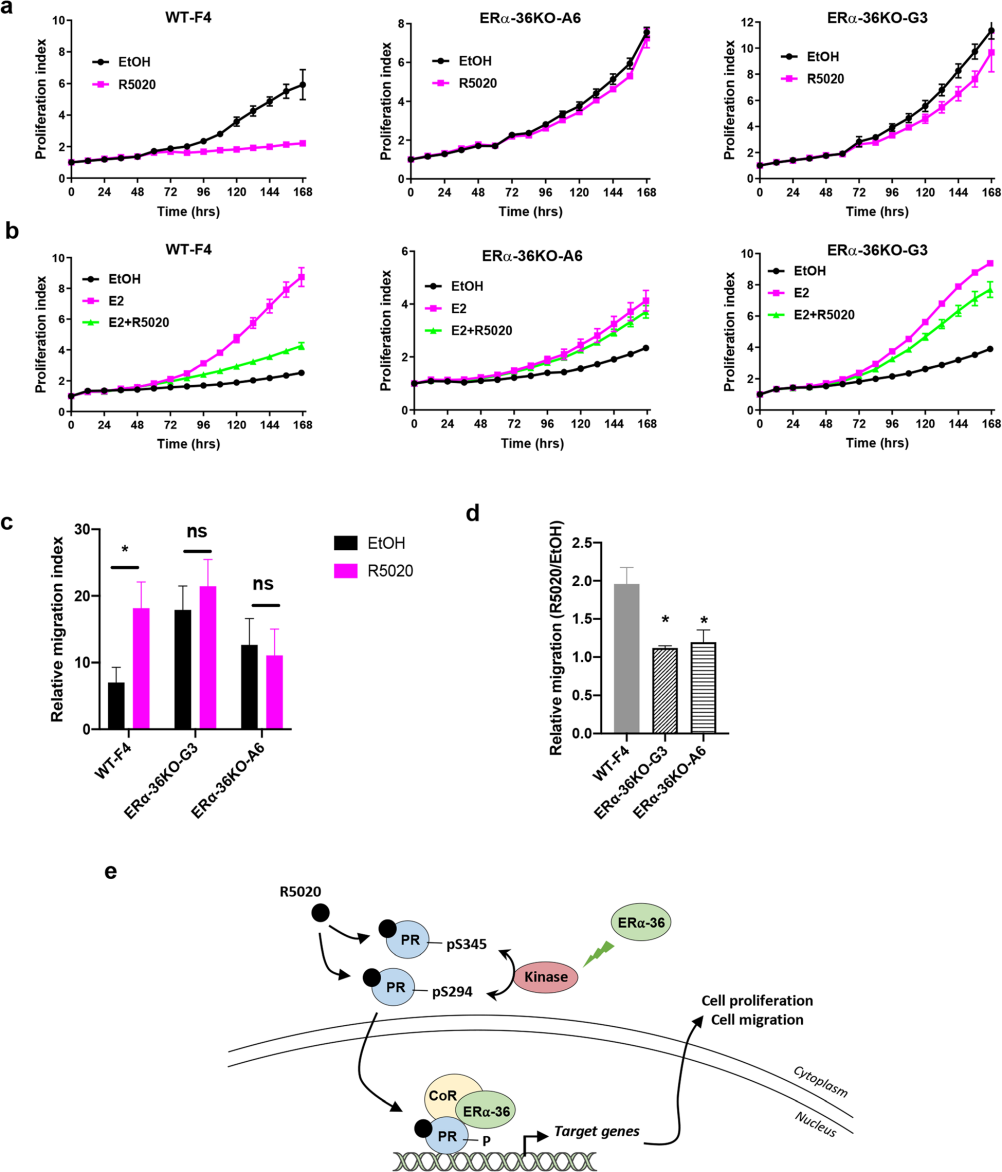
ERα-36 regulates progesterone-mediated cell proliferation. **a** T47D clones were plated onto 96-well plates and treated with R5020 (10 nM) or ethanol, and proliferation was measured using the IncuCyte technology. Image acquisition was conducted every hour using the IncuCyte software, which calculates the percentage of cell confluency as a function of time over 7 days. The results are represented as graphs showing the rate of proliferation every 24 h. The mean ± SD of one experiment representative of three experiments is shown. **b** The same experiment was performed, but the cells were steroid-deprived and treated with E2 (10 nM), R5020 (10 nM), or both. **c** Wound healing assays were performed in T47D clones WT or KO for ERα-36 as described in the “Methods” section. The percent of migration was determined as the mean of the distance of the wound for the different experiments. The analysis was performed in three separate experiments. Data are represented as means ± SEM from three replicates in each of the three independent experiments. *p < 0.05; ns, non-significant. **d** Using the wound healing assay performed in **c**, the relative migration R5020-dependent was calculated as the mean ± SEM for the three independent experiments. *p < 0.05. **e** Model of ERα-36 regulation of PR signaling. Upon progesterone treatment, ERα-36 activates a kinase involved in the phosphorylation of PR at S294 and S345 residues. ERα-36 could participate in coregulator recruitment to regulate PR transcriptional activity in a gene-dependent manner, which in turn modulates cell proliferation and migration. CoR, coregulators

The effects of ERα-36 on cell proliferation were next assessed using the IncuCyte technology. We found that ERα-36 has no major impact on the proliferation of T47D cells (Additional file 6). Although numerous studies have shown that PR is an important actor of breast tumorigenesis and progression [18–20], progesterone can also inhibit FBS- and E2-induced cell proliferation [21–23]; we wondered whether ERα-36 was involved in this process. Of note, we found that the inhibitory effect of R5020 on serum-induced proliferation was lost in ERα-36 KO compared to WT clones (Fig. 6a). Interestingly, ERα-36 was also involved in R5020 regulation of E2-induced proliferation. Indeed, while R5020 inhibited the effects of E2 on cell growth in WT cells, this effect on cell proliferation was abolished in ERα-36 KO cells (Fig. 6b). The mechanism seems to be independent of ERα/PR interaction since ERα-36 KO does not impair their interaction (Additional file 7). Next, we investigated whether ERα-36 played a role in the effect observed for progesterone on cell migration. We found that ERα-36 KO significantly impeded cell migration of T47D cells in the presence of R5020 (Fig. 6c, d).

Altogether, these results clearly demonstrate that ERα-36 is involved in PR expression, signaling, and transcriptional activity, highlighting a new regulator of progesterone signaling.

Figure 6e summarizes a model established according to our findings presenting the mechanisms of action of ERα-36 on progesterone signaling. Consistently, this splice variant of ERα regulates PR phosphorylation by participating in the activation of an as yet unidentified kinase, and is also involved in its transcriptional activity, modulating the expression of genes participating in progesterone-mediated cell proliferation and migration.

## Discussion

ERα-36, the well-characterized splice variant of ERα plays an important role in breast tumorigenesis, and its expression has been associated with poor patient survival, owing primarily to its involvement in tamoxifen resistance and metastasis development [11]. However, its prognostic value has as yet not been studied in different BC subtypes. In this work, based on a cohort of breast cancer patients, we analyzed the expression of ERα-36 alongside patient outcome and traditional prognostic markers and reveal that its poor predictive value is significantly associated with PR-positive tumors. Moreover, we identify ERα-36 as an important actor of progesterone signaling, modulating its expression, transcriptional activity, and anti-proliferative and migratory function in breast cancer cells.

We herein found that while ERα-36 is weakly expressed in the cytoplasm of almost all tumors, its membrane expression occurs only in 40% of breast tumors independently of ERα and PR status. These results corroborate previous studies on ERα-36 expression in BC [11, 14]. In addition, its expression was associated with a high SBR grade and a decrease in patient survival in terms of OS, DFS, and DMFS, supporting published results showing that ERα-36 is associated with the development of metastases [11]. Of interest, we determined that its prognostic value is significant in PR-positive tumors versus PR-negative tumors, suggesting that ERα-36 could interfere with PR signaling. This observation, despite based on a retrospective analysis of a single cohort, is a door opener to dissect the details of the interaction between the 2 proteins. By several approaches, we clearly demonstrated that ERα-36 binds to PR. Interestingly, its C-terminal domain is not involved in this interaction, indicating that it interacts with PR via a domain shared with ERα. We also identified that the interaction between ERα-36/PR occurs via its binding to 2 domains of PR, namely PR3 and PR5, containing the DBD and the LBD, respectively. The binding sites are different from those of ERα, as it binds to 2 sites within the PR sequence located within PR1 and PR2 domains [24]. This may explain why ERα-36 KO does not impede ERα binding to PR (Sup. Fig. 3).

Interestingly, although ERα-36 is mainly localized in the cytoplasm and at the plasma membrane of cells, it interacts with PR exclusively in the nucleus of cells, suggesting that ERα-36 could regulate the transcriptional activity of PR. Indeed, ERα-36 has already been shown to regulate the transcription of *ALDH1A1* by binding to its promoter [11].

We also showed that ERα-36 regulates PR expression at the level of the mRNA. The low level of ERα recruitment on PR promotor in T47D cells did not allow to conclude whether this effect is mediated through ERα. As miRNAs have been shown to control PR expression [25, 26], we can hypothesize that ERα-36 could regulate miRNA expression to modulate PR level within the cells.

Interestingly, we found that phosphorylation of PR on S294 and S345 strongly decreased in cells KO for ERα-36, indicating that ERα-36 may regulate the expression and/or activity of kinases. However, although ERK was described to phosphorylate these 2 residues, the kinase is not involved in our present study as p-ERK was not modified in cells KO for ERα-36, although PR phosphorylation strongly decreased, and the ERK inhibitor did not change the phosphorylation status of PR (Sup. Fig. 2).

Given the fact that ERα-36 binds PR in the nucleus and that S294 and S345 are involved in the transcriptional activity of PR [27–29], we also assessed whether ERα-36 could regulate PR-mediated transcription. A luciferase assay confirmed that ERα-36 activates the transcriptional activity of PR on an artificial promoter and is involved in the expression of several PR target genes, including DUSP1, RGS2, and PDK4 (downregulated); SGK1 (upregulated); and FKBP5 (unchanged). However, Chip experiments showed that PR binding remains constant for the genes tested. As we found that ERα-36 binds to the E domain of PR, which contains binding sites for coregulators, we can hypothesize that ERα-36 could modulate the binding of coregulators in a gene-dependent manner.

We also assessed whether ERα-36 could play a role in the effects of progesterone on cell proliferation. The role of progesterone in breast tumorigenesis is complex as there is a differential effect of PR in normal and malignant breast tissue [30]. Although administration of PR agonist MPA to mice promotes the formation of mammary tumors initiated by DMBA [31], it exerts a biphasic response in cell lines, such as a rapid proliferation burst followed by a sustained growth arrest [32–34]. More recently, several articles clearly showed that in addition to proliferative action, under certain circumstances, progesterone has also an anti-proliferative action in cellulo and in vivo [22, 34, 35]. Interestingly, although ERα-36 has no striking effect on cell growth, we found that its depletion abolished the inhibitory effect of progesterone on FBS- and E2-dependent cell proliferation. As Carroll’s team demonstrated that this effect involves PR/ERα interaction [22], we investigated whether this interaction is modified in cells knock-out for ERα-36, but no difference was observed, suggesting that other mechanisms of regulation may be involved. Indeed, Sartorius’s team found that the inhibitory effect of progesterone on cell proliferation is largely due to a direct binding of PR to the RNA polymerase III, regulating tRNA transcription affecting gene sets at the translational level [35]. More recently, Vicent’s team showed that progesterone negatively regulates cell proliferation via a functional crosstalk with the transcription factor C/EBPα [34]. Interestingly, we found that the progesterone-induced C/EBPα expression was dependent on ERα-36. In addition, DUSP1 was reported to mediate inhibitory effects of progesterone on cell proliferation [21, 34], and our present work describes that ERα-36 KO also inhibit progesterone-induced DUSP1 transcription, potentially explaining how progesterone fails, at least in part, to inhibit FBS- and E2-dependent cell proliferation in ERα-36 KO cells.

In conclusion, we demonstrate herein that ERα-36 is involved in progesterone anti-proliferative and migratory effects. This latter could explain why in our study ERα-36 expression in PR-positive tumors is associated with a reduced distant metastasis-free survival.

Altogether, our present data show that ERα-36 is a new regulator of PR, concomitantly acting on its expression and its activity. Further studies are required to validate its use as a new biomarker for a subset of PR-positive tumors with poor prognosis.

## Conclusions

In conclusion, we confirmed that ERα36 expression at the plasma membrane is a marker of poor prognosis. However, its correlation with patient survival is only observed in PR-positive tumors.

We also found that ERα36 regulates the signaling and transcriptional activity of progesterone in cellulo. Moreover, ERα36 is required for allowing the anti-proliferative effect of progesterone as well as its role in cell migration. Further studies are required to decipher whether other biological processes are altered.

## Supplementary information

**Additional file 1.** : List of the primers used for RT-PCR experiments.

**Additional file 2.** : List of the primers used for Chip-qPCR.

**Additional file 3.** : Study of ERα binding to PR regulatory sequences.

**Additional file 4.** : Effect of ERK inhibitor on PR phosphorylation.

**Additional file 5.** : ERα-36 regulates PR transcriptional activity.

**Additional file 6.** : ERα-36 does not modify T47D cell proliferation.

**Additional file 7.** : Study of PR/ERα interaction.

## Data Availability

All data are available within the article. All materials are available from the authors upon request.

## Abbreviations

BC: Breast cancer
ERα: Estrogen receptor α
PR: Progesterone receptor
DMFS: Distant metastasis-free survival
DFS: Disease-free survival
OS: Overall survival
Tam: Tamoxifen
AI: Aromatase inhibitor
Anthra: Anthracycline
PLA: Proximity ligation assay
Eth: Ethanol
