# Supplementary material for: ERα-36 regulates progesterone receptor activity in breast cancer
Source: Breast Cancer Res. 2020 May 19;22:50. doi: 10.1186/s13058-020-01278-7 (PMC7238515; doi:10.1186/s13058-020-01278-7)
Supplement: Supplementary file 1 — Additional file 1. : List of the primers used for RT-PCR experiments. [file 13058_2020_1278_MOESM1_ESM.docx]

**Additional File 1**: List of the primers used for RT-qPCR experiments

| **Target genes** | **Sequence (forward)** | **Sequence (reverse)** |
| --- | --- | --- |
| **28S** | 5’-CGATCCATCATCCGCAATG-3’ | 5’-AGCCAAGCTCAGCGCAAC-3’ |
| **DUSP1** | 5’-CCTGCAGTACCCCACTCTACG-3’ | 5’-CCCAAGGCATCCAGCATGTCC-3’ |
| **RGS2** | 5’-AGCTGTCCTCAAAAGCAAGG-3’ | 5’-TCTGGGCAATCAGAGTTTTG-3’ |
| **PDK4** | 5’-CATACTCCACTGCACCAACG-3’ | 5’-AGAAATTGGCAAGCCGTAAC-3’ |
| **SGK1** | 5’-GCAGAAGAAGTGTTCTATGCAGT-3’ | 5’-CCGCTCCGACATAATATGCTT-3’ |
| **STAT5A** | 5’-AAGCCCCACTGGAATGATGG-3’ | 5’-GGAGTCAAACTTCCAGGCGA-3’ |
| **FKBP5** | 5’-GGATATACGCCAACATGTTCAA-3’ | 5’-CCATTGCTTTATTGGCCTCT-3’ |
| **C/EBP** | 5’ TGG ACA AGA ACA GCA ACG AG 3’ | 5’AGC TCC AGC ACC TTC TGC T3’ |
