# Supplementary material for: ERα-36 regulates progesterone receptor activity in breast cancer
Source: Breast Cancer Res. 2020 May 19;22:50. doi: 10.1186/s13058-020-01278-7 (PMC7238515; doi:10.1186/s13058-020-01278-7)
Supplement: Supplementary file 2 — Additional file 2. : List of the primers used for Chip-qPCR. [file 13058_2020_1278_MOESM2_ESM.docx]

**Additional File 2**: List of the primers used for ChIP-qPCR

| **Response Elements** | **Sequence (forward)** | **Sequence (reverse)** |
| --- | --- | --- |
| **PgR promoter** | 5’-AGGGAGGAGAAAGTGGGTGT-3’ | 5’-GGAGAACTCCCCGAGTTAGG-3’ |
| **PgR Enhancer 1** | 5’-GCCTGACCTGTTGCTTCAAT-3’ | 5’-GCAGGACGACTTCTCAGACC-3’ |
| **PgR Enhancer 2** | 5’-AACGTGTTTGCATCTTGCTG-3’ | 5’-GGGCTGGCTTTTTATCATTCA-3’ |
| **DUSP1** | 5’-ATCGCAACACTTGGGAAGAC-3’ | 5’-AACCGCAGAATGTTCCTGAC-3’ |
| **PDK4 (-815)** | 5’-CACATGGCAGGAACTGTACG-3’ | 5’-CGTACAGGGCAGAGTGAACA-3’ |
| **SGK1** | 5’-TCAATTGTCGTGCTCAAAGG-3’ | 5’-AGGCAAGAACAGGGAAGTCA-3’ |
| **STAT5A** | 5’-GGACTACTGTGAATTGGCTCGT-3’ | 5’-GCTTTCTGTTTCTGTTCCTTGA-3’ |
| **FKBP5** | 5’-AACACCCTGTTCTGAATGTGG-3’ | 5’-GCATGGTTTAGGGGTTCTTG-3’ |
