# Supplementary material for: ERα-36 regulates progesterone receptor activity in breast cancer
Source: Breast Cancer Res. 2020 May 19;22:50. doi: 10.1186/s13058-020-01278-7 (PMC7238515; doi:10.1186/s13058-020-01278-7)
Supplement: Supplementary file 3 — Additional file 3. : Study of ERα binding to PR regulatory sequences. [file 13058_2020_1278_MOESM3_ESM.docx]

**Additional File 3: Study of ERα binding to PR regulatory sequences**

MCF-7 and T47D cells were grown in charcoal-stripped serum for 48 h and then treated with 10 nM E2 for 2 h, were subjected to ChIP assay using anti-ERα antibody. The precipitated DNA fragments were used for qPCR analysis using specific primers for the indicated promoters. Results are expressed relative to the signal obtained from input chromatin.
