# Supplementary figures and images for: ERα-36 regulates progesterone receptor activity in breast cancer
Source: Breast Cancer Res. 2020 May 19;22:50. doi: 10.1186/s13058-020-01278-7 (PMC7238515; doi:10.1186/s13058-020-01278-7)

**
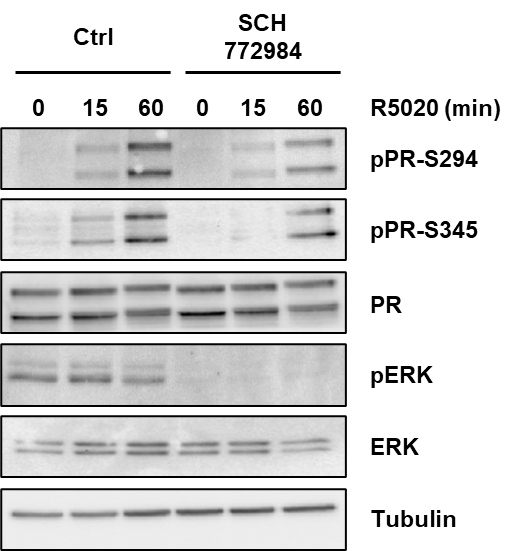
**

**Additional File 4: Effect of ERK inhibitor on PR phosphorylation**

Supplement: Supplementary file 4 — Additional file 4. : Effect of ERK inhibitor on PR phosphorylation. [file 13058_2020_1278_MOESM4_ESM.docx]
