# Supplementary material for: ERα-36 regulates progesterone receptor activity in breast cancer
Source: Breast Cancer Res. 2020 May 19;22:50. doi: 10.1186/s13058-020-01278-7 (PMC7238515; doi:10.1186/s13058-020-01278-7)
Supplement: Supplementary file 5 — Additional file 5. : ERα-36 regulates PR transcriptional activity. [file 13058_2020_1278_MOESM5_ESM.docx]

**Additional File 5: ERα-36 regulates PR transcriptional activity**
