# Supplementary material for: ERα-36 regulates progesterone receptor activity in breast cancer
Source: Breast Cancer Res. 2020 May 19;22:50. doi: 10.1186/s13058-020-01278-7 (PMC7238515; doi:10.1186/s13058-020-01278-7)
Supplement: Supplementary file 6 — Additional file 6. : ERα-36 does not modify T47D cell proliferation. [file 13058_2020_1278_MOESM6_ESM.docx]

**Additional File 6: ERα-36 does not modify T47D cell proliferation**
