# Supplementary material for: ERα-36 regulates progesterone receptor activity in breast cancer
Source: Breast Cancer Res. 2020 May 19;22:50. doi: 10.1186/s13058-020-01278-7 (PMC7238515; doi:10.1186/s13058-020-01278-7)
Supplement: Supplementary file 7 — Additional file 7. : Study of PR/ERα interaction. [file 13058_2020_1278_MOESM7_ESM.docx]

**Additional File 7: Study of PR/ERα interaction**

Cell extracts from T47D WTF4 and ERα-36KOA6 and G3 were immunoprecipitated with PR or irrelevant antibodies. Western blotting was then used to evaluate ERα interaction (left-hand panel). ERα, PR and GAPDH expression was studied in the inputs (middle panel). Quantification of IP results was performed (right-hand panel).
